# Pembrolizumab in combination with gemcitabine for patients with HER2-negative advanced breast cancer: GEICAM/2015–04 (PANGEA-Breast) study

**DOI:** 10.1186/s12885-022-10363-3

**Published:** 2022-12-03

**Authors:** L. de la Cruz-Merino, M. Gion, J. Cruz, JL. Alonso-Romero, V. Quiroga, F. Moreno, R. Andrés, M. Santisteban, M. Ramos, E. Holgado, J. Cortés, E. López-Miranda, A. Cortés, F. Henao, N. Palazón-Carrión, L. M. Rodriguez, I. Ceballos, A. Soto, A. Puertes, M. Casas, S. Benito, M. Chiesa, S. Bezares, R. Caballero, C. Jiménez-Cortegana, V. Sánchez-Margalet, F. Rojo

**Affiliations:** 1 Department of Medical Oncology, Medicine Department, Virgen Macarena University Hospital, University of Seville, Dr. Fedriani St, No. 3, Seville, 41009 Spain; 2 GEICAM Spanish Breast Cancer Group, San Sebastián de los Reyes, Madrid, Spain; 3 Department of Medical Oncology, Hospital Universitario Ramón y Cajal, Madrid, Spain; 4 Department of Medical Oncology, Hospital Universitario de Canarias, Santa Cruz de Tenerife, Spain; 5 Department of Medical Oncology, Hospital Clínico Universitario Virgen de La Arrixaca-IMIB, Murcia, Spain; 6 Department of Medical Oncology, Badalona Applied Research Group in Oncology (B-ARGO Group), Catalan Institute of Oncology, Badalona, Spain; 7 Department of Medical Oncology, Hospital Clínico Universitario San Carlos, Madrid, Spain; 8 Department of Medical Oncology, Hospital Clínico Universitario Lozano Blesa, Saragossa, Spain; 9 Department of Medical Oncology, Clínica Universidad de Navarra, Navarra, Spain; 10 IdiSNA, Navarra Institute for Health Research, Pamplona, Spain; 11 Department of Medical Oncology, Centro Oncológico de Galicia, A Coruña, Spain; 12 Department of Medical Oncology, Hospital La Luz, Quironsalud, Madrid, Spain; 13 International Breast Cancer Center (IBCC), Quiron Group, Barcelona and Madrid, Spain; 14 Vall d´Hebron Institute of Oncology (VHIO), Barcelona, Spain; 15 Faculty of Biomedical and Health Sciences, Department of Medicine, Universidad Europea de Madrid, Madrid, Spain; 16 Medical Biochemistry and Molecular Biology and Immunology Department, Virgen Macarena University Hospital, University of Seville, Seville, Spain; 17 Pathology Department, IIS-Fundación Jiménez Díaz, Madrid, Spain; 18 CIBERONC-ISCIII, Madrid, Spain

**Keywords:** Pembrolizumab, Chemotherapy, HER2-negative, Advanced breast cancer, TILs, PD-L1, MDSCs

## Abstract

**Background:**

We evaluated a new chemoimmunotherapy combination based on the anti-PD1 monoclonal antibody pembrolizumab and the pyrimidine antimetabolite gemcitabine in HER2- advanced breast cancer (ABC) patients previously treated in the advanced setting, in order to explore a potential synergism that could eventually obtain long term benefit in these patients.

**Methods:**

HER2-negative ABC patients received 21-day cycles of pembrolizumab 200 mg (day 1) and gemcitabine (days 1 and 8). A run-in-phase (6 + 6 design) was planned with two dose levels (DL) of gemcitabine (1,250 mg/m^2^ [DL0]; 1,000 mg/m^2^ [DL1]) to determine the recommended phase II dose (RP2D). The primary objective was objective response rate (ORR). Tumor infiltrating lymphocytes (TILs) density and PD-L1 expression in tumors and myeloid-derived suppressor cells (MDSCs) levels in peripheral blood were analyzed.

**Results:**

Fourteen patients were treated with DL0, resulting in RP2D. Thirty-six patients were evaluated during the first stage of Simon’s design. Recruitment was stopped as statistical assumptions were not met. The median age was 52; 21 (58%) patients had triple-negative disease, 28 (78%) visceral involvement, and 27 (75%) ≥ 2 metastatic locations. Progression disease was observed in 29 patients. ORR was 15% (95% CI, 5–32). Eight patients were treated ≥ 6 months before progression. Fourteen patients reported grade ≥ 3 treatment-related adverse events. Due to the small sample size, we did not find any clear association between immune tumor biomarkers and treatment efficacy that could identify a subgroup with higher probability of response or better survival. However, patients that experienced a clinical benefit showed decreased MDSCs levels in peripheral blood along the treatment.

**Conclusion:**

Pembrolizumab 200 mg and gemcitabine 1,250 mg/m^2^ were considered as RP2D. The objective of ORR was not met; however, 22% patients were on treatment for ≥ 6 months. ABC patients that could benefit of chemoimmunotherapy strategies must be carefully selected by robust and validated biomarkers. In our heavily pretreated population, TILs, PD-L1 expression and MDSCs levels could not identify a subgroup of patients for whom the combination of gemcitabine and pembrolizumab would induce long term benefit.

**Trial registration:**

ClinicalTrials.gov and EudraCT (NCT03025880 and 2016–001,779-54, respectively). Registration dates: 20/01/2017 and 18/11/2016, respectively.

## Background

Immunotherapy based on immune checkpoint inhibitors (ICIs) has revolutionized treatment of several cancer types. An impact on long-term benefit and overall survival (OS) is documented in the advanced setting of several tumors treated with ICIs [1–3]. However, the potential role of immunotherapy in breast cancer (BC) has been largely discussed, as BC has been considered traditionally a “cold” non-immunogenic tumor. At this point, efforts have been focused on the triple-negative (TN) BC subtype, as it shows higher levels of tumor infiltrating lymphocytes (TILs) and programmed death-ligand 1 (PD-L1) in its tumor microenvironment [4–6], which are currently suggested as the best predictive immune biomarkers to guide immunotherapy in BC. Recently, two phase III clinical trials [7–9] reported positive results in PD-L1 + metastatic TNBC patients. However, other trials [10] with similar designs have reported negative outcomes. Therefore, the real value of immunotherapy for BC, and especially for TNBC, needs additional studies, with new combinations and translational approaches identify clearer which patients are candidates for this strategy.

In this trial, we aimed to test whether the combination of gemcitabine and pembrolizumab could be feasible and provide meaningful responses in pretreated advanced BC (ABC) patients with TNBC or luminal A/B subtype according to St. Gallen recommendations [11], irrespective of their PD-L1 status. Pembrolizumab is a humanized immunoglobulin (Ig) G4 kappa PD-1 monoclonal antibody (mAb) that blocks the inhibitory T-cell signaling induced by the PD1/PD-L1 axis, preserving the antitumor activity of specific cytotoxic T cells. On the other side, gemcitabine is a nucleoside analogue of cytarabine, a pyrimidine antimetabolite, that interferes with the DNA synthesis by different mechanisms. In addition, gemcitabine seems to exert indirect immunogenic effects inducing tumor cell apoptosis that increase antigen cross-presentation, and enhancing CD8 + T cell and natural killer (NK) cell-mediated anti-tumor immunity through elimination of myeloid-derived suppressor cells (MDSCs) and regulatory T cells [12–16].

Although in the clinical setting gemcitabine activity in ABC is modest at best, it is a well-tolerated treatment in most of the cases [17, 18], as it is pembrolizumab, and therefore a combination of both compounds, in the search of a potential synergism was planned in this trial. An initial run-in phase was conceived to reassure preliminary safety assumptions, as experience with this combination at that moment was limited.

## Methods

### Study design

The PANGEA-Breast was an open-label, single-arm, multicenter phase II trial conducted in Spain. Gemcitabine plus pembrolizumab was evaluated in human epidermal growth factor receptor 2 (HER2)-negative ABC patients, with balanced distribution between TN and hormone receptor-positive (HR +) cohorts. In an initial exploratory run-in-phase with a 6 + 6 design, toxicity was evaluated within the first cycle. Fixed doses of pembrolizumab on day 1 and gemcitabine on days 1 and 8 of each 21-day cycle were administered to determine the recommended phase II dose (RP2D) based on the occurrence of any dose-limiting toxicity (DLT). Dose level (DL) 0 comprised pembrolizumab 200 mg and gemcitabine 1,250 mg/m^2^ as an intravenous (IV) infusion. De-escalation to DL-1 (gemcitabine 1,000 mg/m^2^) was planned if DL0 was not tolerable.

Once the RP2D was defined, eligible patients were enrolled in phase II. The primary objective was the objective response rate (ORR), defined as the number of patients with ≥ 1 treatment dose and complete response (CR) plus partial response (PR) according to the Response Evaluation Criteria in Solid Tumors (RECIST) version 1.1. The secondary objectives were clinical benefit rate (CBR) with stable disease (SD) of ≥ 24 weeks, duration of response (DoR), progression-free survival (PFS), OS, and safety and tolerability according to the National Cancer Institute Common Terminology Criteria for Adverse Events (NCI-CTCAE) version 4.0. The exploratory objectives were to assess the efficacy based on immune-related response criteria, search for tumor tissue and peripheral blood biomarkers for clinical activity, correlate a set of immune biomarkers with disease evolution and efficacy of the combination, and comparison of this set of biomarkers from cohorts of healthy volunteers and patients from this trial.

The study was conducted according to the International Conference on Harmonization Good Clinical Practice Guidelines and Declaration of Helsinki and was approved by the institutional ethical review boards of the participating sites and Spanish health authorities. It was registered at ClinicalTrials.gov and EudraCT (NCT03025880 and 2016–001,779-54, respectively). Written informed consent was obtained from all patients before performing any protocol-specific procedures.

### Patients

The key inclusion criteria were women aged ≥ 18 years; HER2-negative ABC by immunohistochemistry (IHC) and/or *in situ* hybridization based on local testing of the most recent tumor biopsy; ≥ 10 mm measurable lesion as per the RECIST 1.1; an Eastern Cooperative Oncology Group performance status (ECOG PS) of 0 or 1; prior anthracyclines and taxanes (unless contraindicated), and ≥ 2 endocrine therapy (ET) and ≤ 4 chemotherapy (CT) lines for ABC; patient agreement for a fresh metastatic tumor biopsy at inclusion and progressive disease (PD); adequate organ function; negative pregnancy test for women of child-bearing potential (WCBP) and adequate contraception. Patients with treated brain metastases and stable without steroids were allowed. Healthy controls from the University Hospital Virgen Macarena in Seville, Spain, provided written informed consent to participate in the analysis.

### Treatment plan

Patients received pembrolizumab (200 mg IV) on day 1 and gemcitabine (1,250 mg/m^2^ IV) on days 1 and 8 of each 21-day cycle until objective PD, clinical PD (under investigator’s judgment), unacceptable toxicity, death, or consent withdrawal, whichever occurred first.

### Evaluation procedures

Baseline assessments were performed ≤ 28 days from starting treatment. These included tumor assessment using radiological tests accepted by the RECIST 1.1, standard 12-lead electrocardiogram, hematology, biochemistry, coagulation test, thyroid hormones, urinalysis, physical examination, ECOG PS evaluation, and pregnancy test in WCBP. Tumor assessments were performed every 9 weeks until PD. Adverse events (AEs)were graded using the NCI-CTCAE v4.0. Other safety endpoints included regular monitoring of vital signs and laboratory tests.

### Biomarker analyses

#### TILs and PD-L1 assessments

Available pre-treatment metastatic tumor samples were assessed for TILs density, according to the suggested international guidelines [19]. Cut-offs explored for TILs evaluation were ≥ 5%, ≥ 10%, and ≥ 20%. PD-L1 expression measured using IHC was assessed in 29 pre-treatment metastatic tumor samples using the mAb anti-PD-L1 clone 22C3 (Merck, Kenilworth, NJ). We obtained combined positive scores (CPS) for the assessment of PD-L1 positivity and considered CPS values ≥ 1 as positive (PD-L1 +). We also explored additional CPS cut-offs (≥ 5, ≥ 10, and ≥ 20).

The logistic and Cox regression models were used to evaluate the association of TILs density and PD-L1 expression with treatment efficacy in terms of ORR, CBR (with SD ≥ 24 weeks) and PFS according to the RECIST 1.1.

#### Immunophenotyping of whole peripheral blood using flow cytometry analysis

Blood samples were collected in EDTA-K3 tubes at baseline, before cycles 3 (C3) and 6 (C6), or at the end of treatment (EOT), whichever occurred first, to determine MDSCs counts. Cell populations were determined using flow cytometry of the whole blood using the BD FACSCanto™ system (BD Biosciences, San Jose, CA, USA). Monocytic-MDSCs (M-MDSCs) were defined as CD45 + CD11b + CD33 + HLA-DR − CD14 + CD15 − and granulocytic-MDSCs (G-MDSCs) as CD45 + CD11b + CD33 + HLA-DR − CD14 − CD15 + .

#### Monoclonal antibodies

Antibodies were obtained from Becton Dickinson Immunocytometry Systems (BD Biosciences, CA, USA) and were used at the manufacturer’s recommended concentrations.

### Statistical analyses

Simon’s minimax two-stage design was employed for the phase II part of the study with the option of early stopping owing to lack of response. The sample size was calculated by testing the null hypothesis (H0) that gemcitabine resulted in an ORR of approximately 20%. With the study combination, the alternative hypothesis was 35% (an absolute increase of 15%); with an alpha error of 0.05 and a power of 80%, 53 evaluable patients were required. The first stage should include 31 evaluable patients, and if at least seven presented a response, recruitment would continue to include 53 evaluable patients. The H0 of 20% was rejected if ≥ 16 responses were observed in 53 patients. The SAS Enterprise Guide (version 7.15) was used for all analyses.

## Results

### Patients’ characteristics

**Fig. 1:**
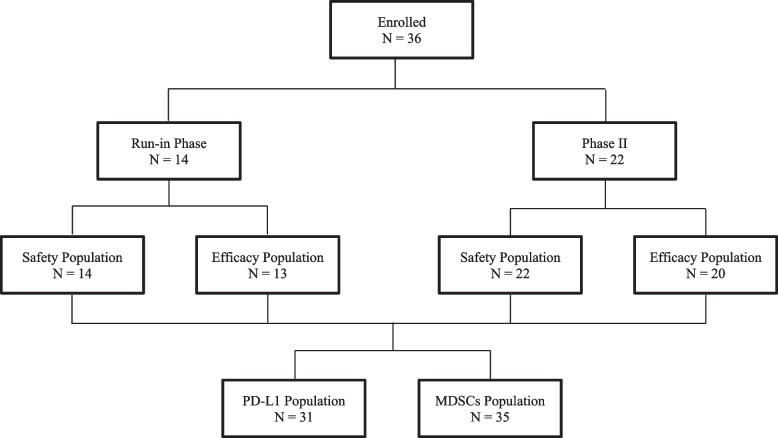
Study flowchart

From June 2017 to May 2018, 36 patients were recruited for the first stage of Simon’s minimax two-stage design (Fig. 1); however, only five patients achieved a response, and recruitment was stopped. Their characteristics are presented in Table 1. Approximately 58% of the tumors were TN. Twenty-three (64%) patients had up to two metastatic locations, and 28 (78%) had visceral involvement. Patients had a median of four prior therapy lines for ABC.

**Table 1:** Patients and disease characteristics

Characteristics	*N* = 36
**Age, years (median [range])**	51 (31–77)
< 65, n (%)	33 (91.7)
≥ 65, n (%)	3 (8.3)
**Menopausal status, n (%)**	
Postmenopausal	26 (72.2)
Premenopausal	10 (27.8)
**ECOG PS, n (%)**	
0	23 (63.9)
1	12 (33.3)
2	1 (2.8)
**Time since 1**^**st**^** BC diagnosis to study inclusion, years (median [range])**	4 (1–37)
**Time since M1 diagnosis to study inclusion, years (median [range])**	2 (0.2–14)
**Number of metastatic locations, n (%)**	
1	9 (25.0)
2	14 (38.9)
≥ 3	13 (36.2)
**Metastatic locations, n (%)**	
Visceral	28 (77.8)
• Liver	25 (69.4)
• Lung	7 (19.4)
• Adrenal gland, pericardial effusion, or pleural involvement	6 (16.6)
Non-visceral only	8 (22.2)
• Bone	20 (55.6)
• Breast	2 (5.6)
• Lymph nodes	13 (36.1)
• Skin	4 (11.1)
• Soft tissue	5 (13.9)
**Histological type, n (%)**	
Invasive ductal carcinoma	33 (91.7)
Invasive lobular carcinoma	2 (5.6)
Invasive squamous carcinoma	1 (2.8)
**Histological grade, n (%)**	
1	1 (2.8)
2	14 (38.9)
3	16 (44.4)
Unknown	5 (13.9)
**HR status (local), n (%)**	
Negative	21 (58.3)
Positive	15 (41.7)
**Ki67 expression (local) (%)**	
Median (range)	30 (15–95)
< 20%, n (%)	5 (13.9)
≥ 20%, n (%)	21 (58.3)
Unknown	10 (27.8)
**BC subtype by IHC (local), n (%)**	
TN	21 (58.3)
HR-positive disease	15 (41.7)
**Prior therapy, n (%)**	
CT	36 (100.0)
• ABC	34 (94.4)
ET	26 (72.2)
• ABC	19 (52.8)
BT	25 (69.4)
• ABC	24 (66.7)
**Number of prior lines for ABC, n (%)**	
None	1 (2.8)
1	9 (25.0)
2	2 (5.6)
3	3 (8.3)
4	5 (13.9)
5	7 (19.4)
≥ 6	9 (25.0)
Median (range)	4 (0–11)
• TN	2 (1–8)
• HR-positive disease	5 (3–11)

*Abbreviations: n* number of patients, *ECOG PS* Eastern Cooperative Oncology Group performance status, *BC* Breast cancer, *M1* Metastases or metastatic disease, *HR* Hormone receptor, *IHC* Immunohistochemistry, *TN* Triple negative, *CT* Chemotherapy, *ET* Endocrine therapy, *BT* Biological therapy, *ABC* Advanced breast cancer

### Run-in phase and determination of RP2D

**Fig. 2:**
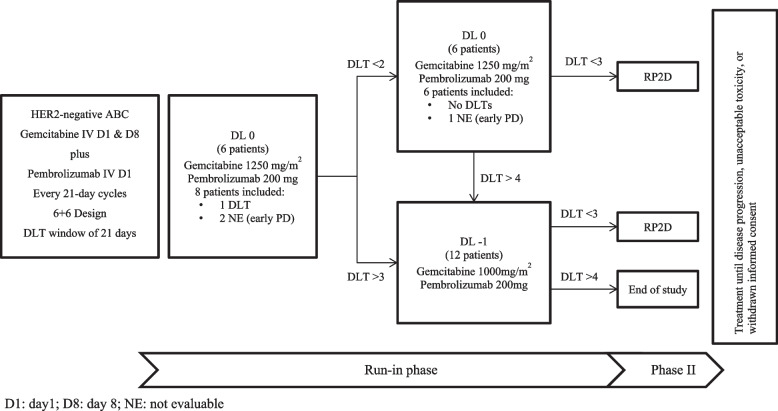
Run-in phase design, patient inclusion, and DLTs

Fourteen patients were treated with DL0. Three patients were replaced since they experienced early PD (Fig. 2). Of the first six patients, one experienced DLT of grade 3 tumor pain and grade 2 anemia and thrombocytopenia; six additional patients were recruited at this DL without any reported DLT; therefore, DL0 was declared as the RP2D.

### Treatment exposure

A median of 4 (range, 1–24) cycles for pembrolizumab and 4.5 (range, 1–24) cycles for gemcitabine were administered. The median relative dose intensity at the RP2D was 100% (100–100) and 80% (39–101), respectively.

Nine (25%) patients experienced delays in pembrolizumab administration because of AEs in five (13.9%) patients; these AEs included hematological and liver function test alterations, nausea, arthralgia, and infections. Thirty (83.3%) patients experienced dose modifications for gemcitabine, with omissions being the most frequent modification followed by reductions and delays. AEs were the most common reasons for all types of modifications. The main reasons for treatment discontinuation included PD in 29 (80.6%) patients; death in two (5.6%); and AE (grade 3 respiratory failure), patient`s decision, and physician’s decision in one (2.8%) patient each. At the time of analysis, two patients were still on treatment.

### Efficacy

**Fig. 3:**
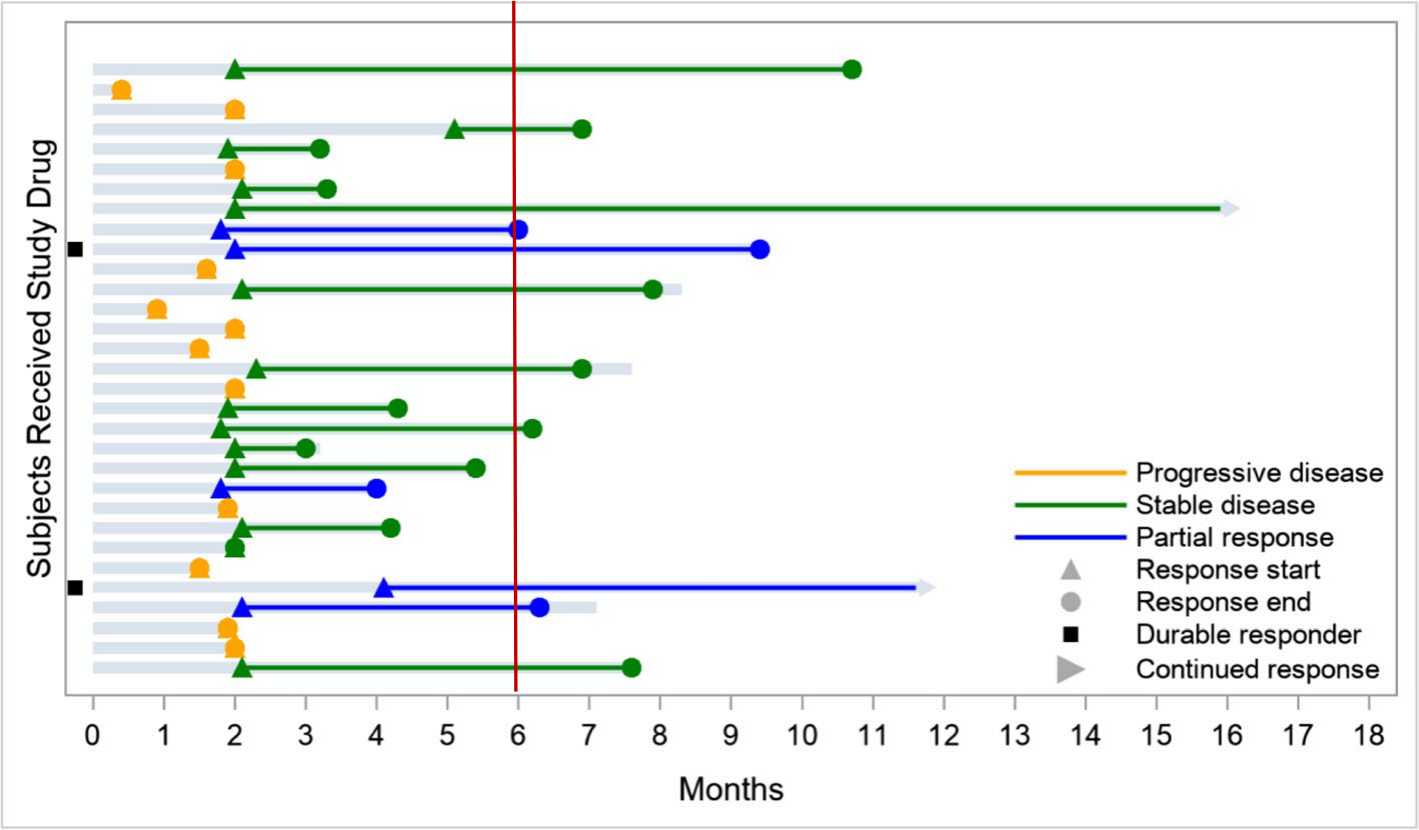
Swimmer plot for the intention-to-treat population

Considering the efficacy population (*n* = 33), the ORR was 15.2% (*n* = 5/33; 95% confidence interval [CI] 5.1–31.9), with only PR reported, and the ORR in patients with TN or HR + BC was similar to the ORR in the whole population. Twelve (36.4%) patients achieved SD (one case lasted for > 6 months), and 12 (36.4%) experienced PD (Fig. 3). The CBR, including SD of any duration, was 51.5% (*n* = 17/33; 95% CI 33.5–69.2). The median DoR was 4.3 months (95% CI 2.3–7.4), median PFS was 3.1 months (95% CI 2.0–4.3), and median OS was 7.9 months (95% CI 6.5–10.3). Eight patients were on treatment for ≥ 6 months before PD (11.4 and 16.1 months in two cases). Efficacy based on immune-related response criteria showed similar results.

### Safety

Almost all patients (35 [97.2%]) experienced AE, which was related to the study treatment in 25 (69.4%) patients. AEs led to treatment discontinuation in five (13.9%) patients. Grade 3 AEs were reported in 15 (41.7%) patients and grade 4 AEs in 7 (19.4%), and of these AEs, their relationship with treatment was established by the investigators in 14 (38.9%) patients. Serious AEs were reported in 12 (33.3%) patients and were related to treatment in three cases. No grade 5 AEs related to treatment were reported. Treatment-emergent AEs, irrespective of their causal relationship with the study treatment, are summarized in Table 2.

**Table 2:** Treatment-emergent adverse events by grade according to the NCI-CTCAE (version 4.03) and with a frequency of at least 5% in any grade

Safety Population (*n* = 36)				
Adverse Event Term	Grade 1, n (%)	Grade 2, n (%)	Grade 3, n (%)	Grade 4, n (%)
Patients with any TEAE	27 (75.0)	27 (75.0)	18 (50.0)	7 (19.4)
Pyrexia	**10 (27.8)**	1 (2.8)	0	0
Fatigue	**11 (30.6)**	10 (27.8)	2 (5.6)	0
Anemia	**8 (22.2)**	2 (5.6)	3 (8.3)	0
Nausea	**6 (16.7)**	2 (5.6)	0	0
Decreased appetite	**4 (11.1)**	1 (2.8)	0	0
Musculoskeletal chest pain	**3 (8.3)**	0	0	0
Pruritus	**3 (8.3)**	0	0	0
Upper respiratory tract infection	**3 (8.3)**	0	0	0
Albuminuria	**2 (5.6)**	0	0	0
Back pain	**2 (5.6)**	0	1 (2.8)	0
Hot flush	**2 (5.6)**	0	0	0
Hypothyroidism	**2 (5.6)**	0	0	0
Abdominal pain upper	1 (2.8)	**2 (5.6)**	0	0
Elevated AST level	1 (2.8)	0	**2 (5.6)**	0
Constipation	1 (2.8)	**2 (5.6)**	1 (2.8)	0
Diarrhea	1 (2.8)	**3 (8.3)**	1 (2.8)	0
Dyspnea	1 (2.8)	**2 (5.6)**	0	0
Painful skin	1 (2.8)	**2 (5.6)**	0	0
Rash	1 (2.8)	**2 (5.6)**	0	0
Tachycardia	1 (2.8)	**2 (5.6)**	0	0
Hypertension	0	**4 (11.1)**	0	0
Neutrophil count decreased	0	6 (16.7)	**10 (27.8)**	3 (8.3)
Weight decreased	0	**2 (5.6)**	0	0

Pembrolizumab-related TEAEs included rash, anemia, decreased neutrophil count, diarrhea, and increased AST and ALT levels

*Abbreviations: TEAE* Treatment-emergent adverse events, *NCI-CTCAE* National Cancer Institute Common Terminology Criteria for Adverse Events, *n* number of patients, *AST* Aspartate aminotransferase

### Association of TILs density and PD-L1 expression with treatment efficacy

**Fig. 4:**
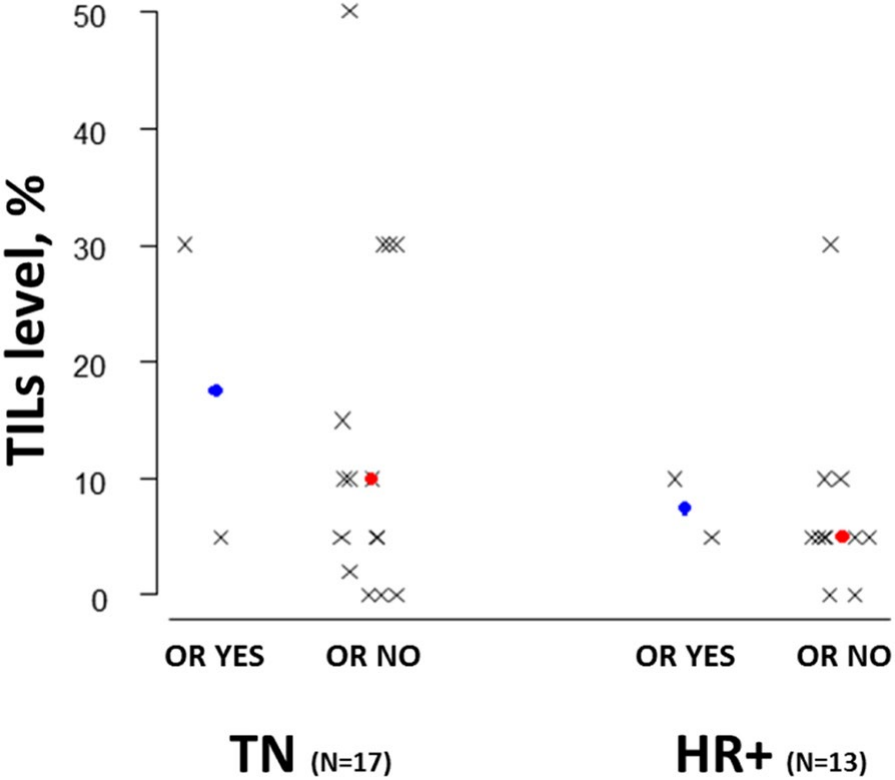
TILs density distribution according to tumor subtype and OR (yes/no)

We evaluated TILs density in 30 (83%) available pre-treatment metastatic tumor samples (Table 3) and correlated these values with clinical endpoints: no significant associations were observed between TILs density and either ORR, CBR or PFS (Table 3). However, analysis of the median TILs density distribution according to tumor subtype showed that TNBC patients who achieved some degree of response tended to present higher levels of lymphocyte infiltration (Fig. 4).

**Table 3:** Logistic and Cox regression models to analyze the association between TILs density and PD-L1 expression (CPS score) with ORR (CR + PR), CBR (CR + PR + SD of > 6 months), and PFS

		**ORR** ***p*-value ** **Odds ratio (95% CI)**	**CBR** ***p*-value ** **Odds ratio (95% CI)**	**PFS** ***p*-value ** **Hazard ratio (95% CI)**
**TILs** **(*n* = 30)** **Cut-Off (n, %)**	**≥5%** (24, 80%) **<5%** (6, 20%)	NA	0.556 0.47 (0.04, 5.90)	0.534 1.37 (0.51, 3.64)
	**≥10%** (13, 43%) **<10%** (17, 57%)	0.773 1.36 (0.17, 11.23)	0.554 0.50 (0.05, 4.98)	0.311 1.51 (0.68, 3.36)
	**≥20%** (6, 20%) **<20%** (24, 80%)	0.789 1.40 (0.12, 16.46)	0.699 0.56 (0.03, 10.93)	0.073 2.50 (0.92, 6.82)
**PD-L1 Score (*n* = 29)** **Cut-Off (n, %)**	**CPS ≥1** (14, 48%) **CPS <1** (15, 52%)	0.941 1.08 (0.13, 8.95)	0.668 1.60 (0.19, 13.69)	**0.038** 2.56 (1.05, 6.23)
	**CPS ≥5** (11, 38%) **CPS <5** (18, 62%)	0.571 0.50 (0.05, 5.51)	0.482 2.50 (0.19, 32.19)	**0.053** 2.34 (0.99, 5.53)
	**CPS ≥10** (8, 28%) **CPS <10** (21, 72%)	0.901 0.86 (0.08, 9.69)	0.793 1.43 (0.10, 20.44)	0.173 1.84 (0.77, 4.42)
	**CPS ≥20** (7, 24%) **CPS <20** (22, 76%)	0.965 1.06 (0.09, 12.14)	0.793 1.43 (0.10, 20.44)	0.072 2.36 (0.93, 6.01)

*Abbreviations: TILs* Tumor-infiltrating lymphocytes, *ORR* Objective response rate, *CBR* Clinical benefit rate, *PFS* Progression-free survival, *CPS* Combined positive score, *CR* Complete response, *PR* Partial response, *SD* Stable disease, *CI* Confidence interval

**Fig. 5:**
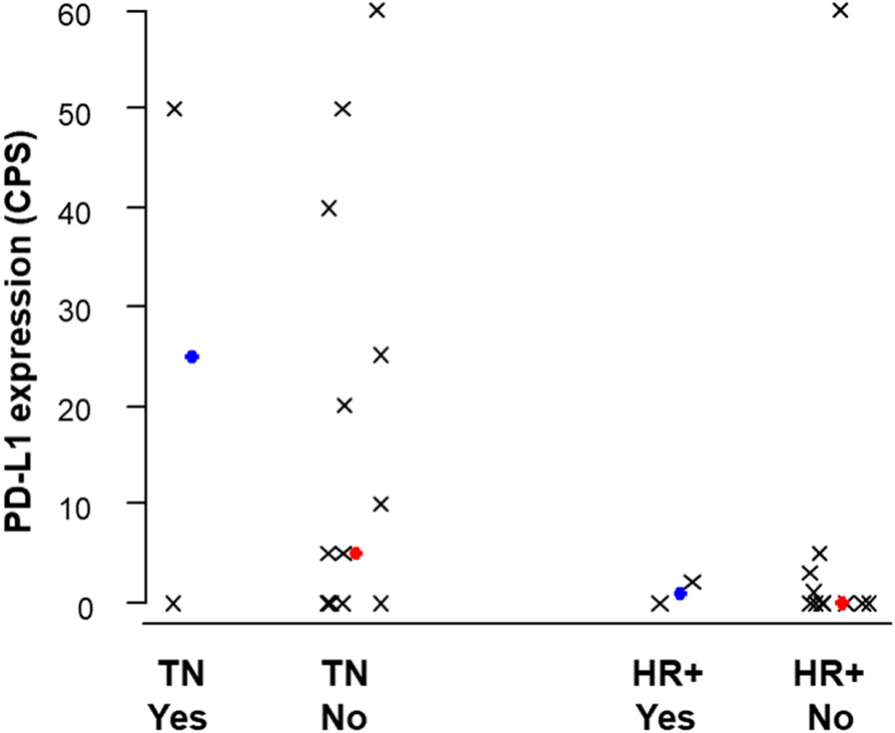
PD-L1 CPS distribution according to tumor subtype and OR (yes/no)

We then assessed PD-L1 expression in 29 (88%) pre-treatment metastatic tumor samples by the established scoring criteria CPS (Table 3): 14 (48%) patients showed PD-L1 CPS scores ≥ 1, thus being considered PD-L1 + . We then associated these scores with clinical endpoints including ORR, CBR and PFS. We observed that patients with CPS values ≥ 1 showed significantly worse PFS (hazard ratio [HR] 2.56; 95% CI 1.05–6.23; *p* = 0.038) and the same trend was observed with CPS values ≥ 5 (HR 2.34; 95% CI 0.99–5.53; *p* = 0.053); this association was not seen with CPS values ≥ 10 and ≥ 20 (Table 3). No association was observed between CPS and the other clinical endpoints ORR and CBR. We then analyzed PD-L1 + scores within different tumor subtypes and correlated them again with clinical endpoints (Fig. 5). We found that 9 (53%) TN patients were PD-L1 + (CPS ≥ 1) Within the HR + subpopulation, 5 (38%) patients were PD-L1 + . PD-L1 + scores were not significantly associated with OR in either TN or HR + .

**Fig. 6:**
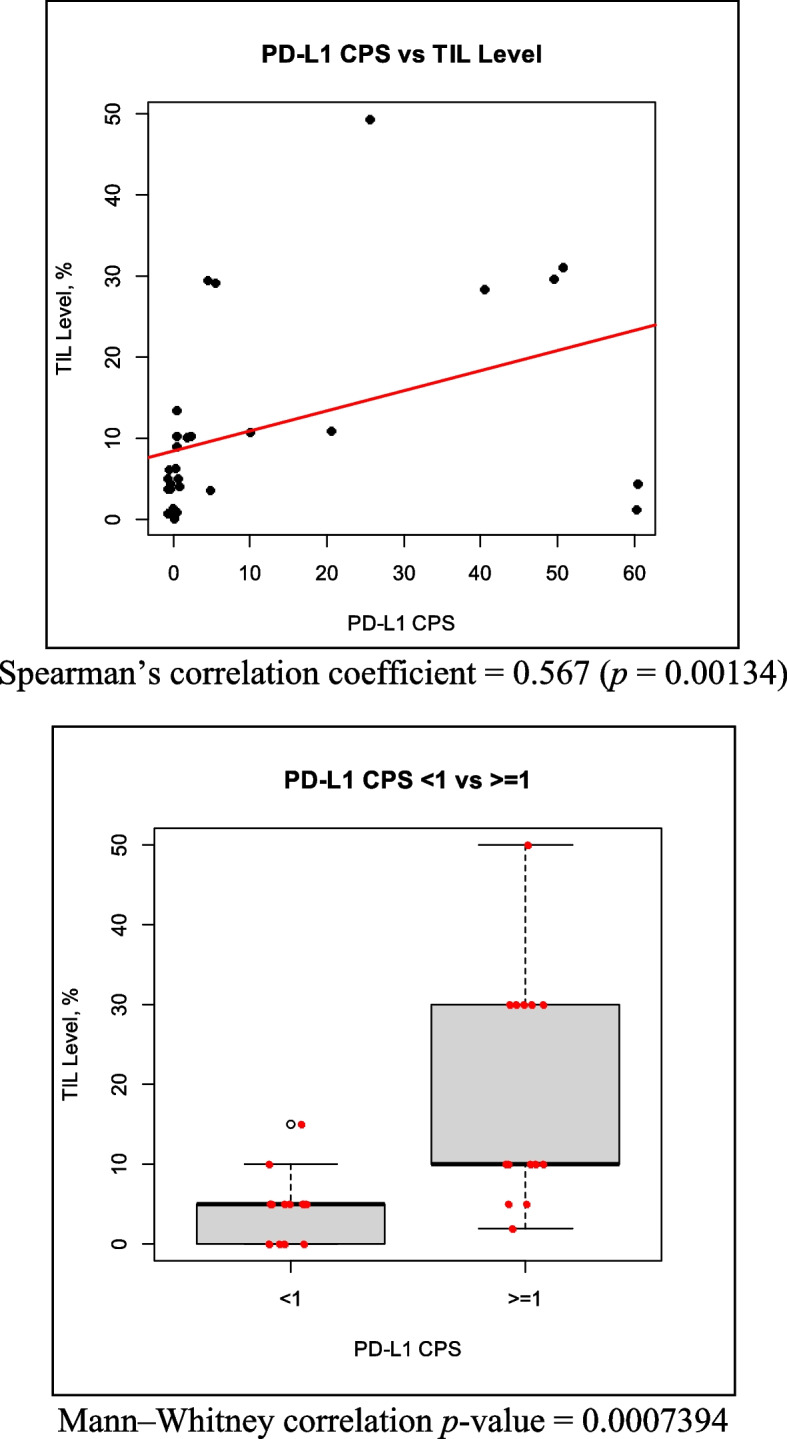
Correlation of TILs density and PD-L1 expression

Finally, we observed a significant positive correlation between TILs levels and PD-L1 expression (correlation coefficient, 0.567; *p* = 0.00134) (Fig. 6).

### Association of MDSCs levels with treatment efficacy

**Fig. 7:**
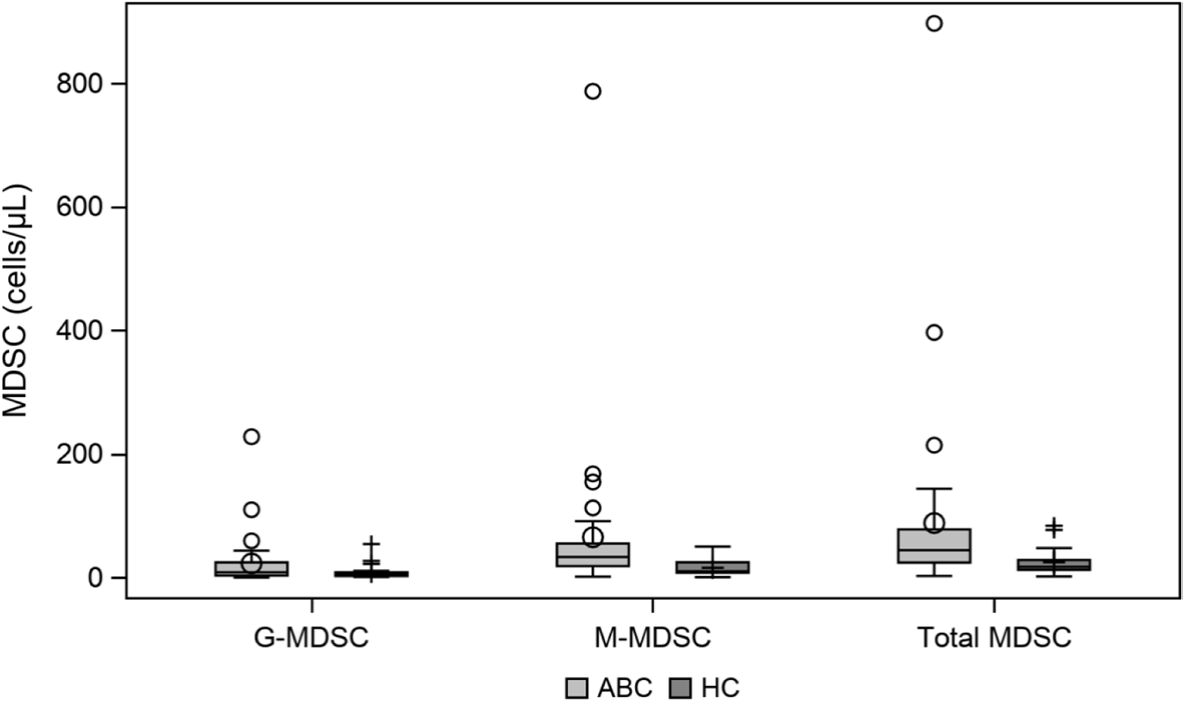
Baseline median values of MDSCs in ABC patients and healthy cohort

Baseline median values of MDSCs were significantly higher in patients vs healthy controls (median values of 44.5 vs. 17.5 cells/µL, respectively, *p* = 0.0018). This difference was especially remarkable in M-MDSCs (median values of 33.5 in patients vs. 10.3 cells/µL in controls, *p* = 0.0010) rather than G-MDSCs (median values of 8.6 vs. 5.0 cells/µl, *p* = 0.1880) (Fig. 7). These differences confirmed a differential immune profile in peripheral blood in healthy donors vs patients at baseline.

Analysis of variation in MDSCs levels along the treatment and correlation with CBR showed a trend towards decreased levels of MDSCs in patients that experienced a clinical benefit (CB) as compared to patients that suffered PD (Table 4). Specifically, patients who obtained CB showed decreased levels of M-MDSCs along the treatment when compared baseline vs. C3 (*p* = 0.0313) and baseline vs. C6/EOT (*p* = 0.0469). Also, when analyzed CB vs PD patients, the first group showed a decrease in G-MDSCs levels at C6/EOT (*p* = 0.0348).

**Table 4:** MDSCs levels (cells/µL) according to tumor response

MDSCs												
	**Total**				**M-MDSCs**				**G-MDSCs**			
	**CB** **(SD ≥ 24 weeks)** **(*n* = 7)**		**PD** **(*n* = 12)**		**CB** **(SD ≥ 24 weeks)** **(*n* = 7)**		**PD** **(*n* = 12)**		**CB** **(SD ≥ 24 weeks)** **(*n* = 7)**		**PD** **(*n* = 12)**	
	**Median**	**IR**	**Median**	**IR**	**Median**	**IR**	**Median**	**IR**	**Median**	**IR**	**Median**	**IR**
**Baseline**	38.6 (*n* = 7)	53.0	47.6 (*n* = 11)	42.8	30.7 (*n* = 7)	32.8	39.8 (*n* = 11)	33.2	7.9 (*n* = 7)	20.9	5.2 (*n* = 11)	27.9
**C3**	17.4 (*n* = 7)	27.0	60.7 (*n* = 11)	247.4	4.3 (*n* = 7)	13.9	47.4 (*n* = 11)	223.1	5.2 (*n* = 7)	10.3	7.7 (*n* = 11)	24.3
**C6/EOT**	12.4 (*n* = 7)	8.5	23.2 (*n* = 5)	37.2	6.3 (*n* = 7)	8.5	8.0 (*n* = 5)	31.2	4.3 (*n* = 7)	10.2	12.4 (*n* = 5)	8.6
**Baseline vs. C6/EOT** ***p-value***	0.0781		0.8750		**0.0469**		0.8750		0.6875		0.6250	
**Baseline vs. C3** ***p-value***	0.2969		0.6953		**0.0313**		0.7695		0.9375		0.6250	
**CB vs. PD** **(C6/EOT)** ***p-value***	0.0513				0.7453				**0.0348**			
**CB vs. PD** **(C3)** ***p-value***	0.1743				0.0572				0.5261			
**CB vs. PD** **(baseline)** ***p-value***	0.3651				0.1743				0.9278			

*Abbreviations: MDSCs* Myeloid derived suppressor cells, *M-MDSCs* Monocytic MDSCs, *G-MDSCs* Granulocytic MDSCs, *CB* Clinical benefit, *SD* Stable disease, *PD* Progressive disease, *IR* Interquartile range, *C3* Cycle 3, *C6* Cycle 6, *EOT* End of treatment

## Discussion

In the PANGEA-Breast trial, pembrolizumab plus gemcitabine achieved a modest ORR of 15.2%. No long-term responders were observed, although two patients were still alive at study closing data (22/07/2021). Some facts may explain these results. First, BC patients included in our study were heavily pretreated in most cases, with a median of four prior lines for ABC. Second, there was no patient preselection with respect to PD-L1 expression or TILs density, which confirms that an unselected population is probably an adverse scenario for immunotherapy in ABC. Finally, gemcitabine probably does not harbor powerful immunogenic properties for treating ABC, as we expected. Therefore, other drugs that induce immunogenic cell death, such as anthracyclines, may be more appropriate. Some clinically outstanding results as those reported in the TONIC trial (35% ORR with doxorubicin [15 mg/m^2^] followed by nivolumab in metastatic TNBC patients) [20] or KEYNOTE-522 study [21] in the neoadjuvant setting, support the hypothesis that the combination of immunotherapy and CT matters, and anthracyclines could trigger and boost the immune response better than other chemotherapeutic drugs. Discordant results between the IMpassion-130 [21] and IMpassion-131 [10] trials can also be partially explained with this theory. The difference among these two trials was the CT used (nab-paclitaxel or conventional paclitaxel, respectively) and premedication with corticosteroids in the IMpassion-131 trial.

Additionally, based on tissue immune biomarkers analysis of the PANGEA-Breast trial, we could not identify a subpopulation benefiting from the chemoimmunotherapy combination. Neither TILs density nor PD-L1 expression using CPS revealed a subgroup with a higher probability of response or better survival. The small sample size (*n* = 36) and highly heterogeneous population (e.g., TN and HR + BC, different previous lines of treatment) represent a major limitation at this point; therefore, our results should be considered cautiously, especially when the IMpassion-130 and KEYNOTE-522 trials have reported favorable results in PD-L1 enriched populations.

Regarding the peripheral blood immune biomarker analysis, MDSCs results were intriguing as their levels were clearly elevated in patients than in the healthy cohort (*p* = 0.0018), suggesting that an immunosuppressive status is induced by ABC. These results are concordant with previous findings that correlate higher levels of MDSCs with adverse prognostic factors and tumor burden in ABC [22]. Interestingly, MDSCs decreased along treatment implementation in the CB group versus PD group with a clear trend at C3 that seem less obvious at C6. MDSCs may represent emerging and valuable biomarkers; however, the limited number of patients and samples in this study jeopardized any major interpretation of our data.

## Conclusions

In summary, this trial reinforces the hypothesis that immunotherapy for ABC could eventually work only in highly selected and enriched populations, ideally for first-line therapy. Pretreated patients, especially those heavily pretreated in our work and in the KEYNOTE-119 trial [23] showed little benefit from ICIs, with a trend of improved efficacy with PD-L1 enrichment. For future clinical trials in this setting, better selection of patients with ABC would be advisable. Additionally, unless new results are available, different original strategies should be tested, as chemoimmunotherapy outcomes in ABC appear globally modest. Approaches aiming to induce the host immune system through effective immunogenic cell death modalities, depletion of immunosuppressive cells such as MDSCs or Tregs [24, 25], or favoring neoantigen presentation [26, 27] could widen the spectrum of immunotherapy for ABC.

## Data Availability

The data that support the findings of this study are available from GEICAM. Data are available from the authors upon reasonable request to the principal investigator Dr. de la Cruz (luis.cruz.sspa@juntadeandalucia.es) and with permission of the PANGEA-Breast steering committee.

## Abbreviations

ABC: Advanced Breast Cancer
AE: Adverse Event
BC: Breast Cancer
C: Cycle
CBR: Clinical Benefit Rate
CPS: Combined Positive Score
CR: Complete Response
CT: Chemotherapy
DL: Dose Levels
DLT: Dose-Limiting Toxicity
DoR: Duration of Response
ECOG PS: Eastern Cooperative Oncology Group Performance Status
EOT: End of Treatment
ET: Endocrine Therapy
G-MDSCs: Granulocytic-MDSCs
H0: Null Hypothesis
HER2: Human Epidermal Growth Factor Receptor 2
HR: Hormone Receptor
HR: Hazard Ratio
IC: Immune Cell
ICIs: Immune Checkpoint Inhibitors
IHC: Immunohistochemistry
IV: Intravenous
mAb: Monoclonal Antibody
MDSC: Myeloid-Derived Suppressor Cells
M-MDSCs: Monocytic-Myeloid-Derived Suppressor Cells
NCI-CTCAE: National Cancer Institute Common Terminology Criteria for Adverse Events
ORR: Objective Response Rate
OS: Overall Survival
PD-L1: Programmed Death-Ligand 1
PFS: Progression-Free Survival
PR: Partial Response
RECIST: Response Evaluation Criteria in Solid Tumors
RP2D: Recommended Phase II Dose
SD: Stable Disease
TILs: Tumor Infiltrating Lymphocytes
TN: Triple-Negative
TNBC: Triple-Negative Breast Cancer
WCBP: Women of Child-Bearing Potential
